# Blink rate and blink timing in children with ADHD and the influence of stimulant medication

**DOI:** 10.1007/s00702-015-1457-6

**Published:** 2015-10-15

**Authors:** Y. Groen, N. A. Börger, J. Koerts, J. Thome, O. Tucha

**Affiliations:** 1 Department of Clinical and Developmental Neuropsychology, Faculty of Behavioural and Social Sciences, University of Groningen, Grote Kruisstraat 2/1, 9712 TS Groningen, The Netherlands; 2 Department of Psychiatry and Psychotherapy, University of Rostock, Gehlsheimer Straße 20, 18147 Rostock, Germany

**Keywords:** Eye blinks, Dopamine, ADHD, Methylphenidate

## Abstract

Spontaneous eye blink rate is modulated by task demands and internal state, and is demonstrated to reflect central dopamine activity. Also, spontaneous eye blinks are strategically timed around salient stimuli. This study investigates whether children with attention deficit hyperactivity disorder (ADHD) show reduced blink rates, blink modulation and blink timing, and whether this is influenced by stimulant medication. The electrooculogram was measured in 18 typically developing children, 16 children with ADHD off methylphenidate (Mph), and 16 children with ADHD on Mph during a rest period and during performance of a 60-min visual selective attention task. Blink rate and timing was extracted from the electrooculogram. No evidence was found for aberrant blink rate or blink modulation in children with ADHD off Mph. All groups increased blink rates from rest to task, and no group differences were found in blink rate during rest and task, or in the modulation of blink rate from rest to task. Time-on task resulted in a similar increase in blink rates in all three groups. Stimulant medication appeared not to influence blink rate and blink modulation, except that in the ADHD off Mph group the blink rate was enhanced only under conditions with performance feedback. All groups inhibited blinks before stimulus presentation and strategically timed their blinks after the stimulus. Children with ADHD off Mph showed reduced blink inhibition before the stimulus; however, given the low incidence (<1 % of the trials) and long latency this is not likely to impair their visual intake.

## Introduction

It is commonly thought that spontaneous eye blinking primarily serves a visual protective function by keeping the eye clean and moist and by protecting it from objects that might injure the eye. However, humans blink 5–10 times more frequently than is necessary to fulfil this function (Karson 1988). This excess of spontaneous blinks has been linked to activity of the central nervous system (see for a review Bacher and Smotherman 2004) and in specific to the activity level of the central dopamine (DA) systems (Karson 1983). A developmental study by Zametkin and colleagues showed that blink rates increase steadily from infancy to adulthood and conclude that blink rate represents a measure of the maturation and integrity of dopaminergic systems in the brain (Zametkin et al. 1979). Adult blink rates in a resting state vary strongly between individuals, ranging from 4 to 48 blinks per minute (mean 14–17 bpm), but within individuals blink rates tend to be remarkably stable (Bentivoglio et al. 1997; Zametkin et al. 1979). Blink rates, however, vary with information processing demands and behavioural states (e.g., relaxation or arousal). In comparison to quiet rest, blink rates increase with activities such as speaking, memorizing and mental arithmetic, and decrease when reading, daydreaming, and performing visually demanding tasks, such as tracking (see for a review Bacher and Smotherman 2004). Blink rates typically increase as a function of time-on task and may therefore reflect the individual’s level of fatigue and the decreased ability to inhibit eye blinking (Stern et al. 1994).

Not only is blink rate modulated by the task demands and behavioural states, but also are eye blinks strategically timed around salient incoming events. Previous studies in healthy children and adults revealed that in visual cognitive tasks blinks are typically inhibited around the presentation of the imperative stimulus until the response is elicited (Pivik and Dykman 2004; Sirevaag et al. 1999). This strategic timing of blinks prevents the loss of relevant information resulting from the visual ‘blackout’ periods caused by the closing of the eyelids, which have been estimated to deprive the visual system from input for at least 200–300 ms (Pivik and Dykman 2004). Eye blinking is therefore not only modulated in a tonic fashion across minutes, but also in a phasic fashion across milliseconds.

There is evidence that spontaneous blink rate is a non-invasive measure of central DA-activity which may provide information about the integrity of the midbrain dopaminergic systems in the brain. Direct evidence for involvement of the central DA systems in eye blinking comes from studies with nonhuman primates, showing that D1 and D2 agonists increase blink rates, which is blocked when the animals were pre-treated with D1 and D2 antagonists (Elsworth et al. 1991). Other evidence comes from psychopathologies associated with hypo- or hyperdopaminergic states. Parkinson’s disease, caused by the progressive loss of nigrostriatal DA producing cells, is associated with reduced rates of spontaneous blinking (Deuschl and Goddemeier 1998). Among patients with schizophrenia, blink rates increase with the number of schizophrenic symptoms and decrease with neuroleptic medication (Karson et al. 1981; Karson 1983; Kleinman et al. 1984).

Attention deficit hyperactivity disorder (ADHD) is also thought to involve dopamine dysfunction, and most theories suggest a hypodopaminergic state in frontostriatal brain areas (Levy and Swanson 2001; Oades et al. 2005). However, the available studies on blink rates of individuals with ADHD have provided mixed evidence for these theories. Three studies, using tasks lasting between 1 and 10 min, failed to find overall differences in blink rates between controls and ADHD children (Caplan et al. 1996; Daugherty et al. 1993; Jacobsen et al. 1996). One study demonstrated a reduced blink rate in children with ADHD compared to controls during a waiting situation of 5 min (Konrad et al. 2003), while another study found no blink rate differences between children with ADHD and controls. This latter study, however, revealed an increased blink rate in boys with ADHD after an intensive treadmill walk (Tantillo et al. 2002). Increased blink rates have recently also been observed in adults with ADHD during a continuous performance test of 20 min, with faster increments with time-on task in adults with ADHD compared to controls (Fried et al. 2014). The study by Caplan et al. (1996) found specific task effects, namely a reduced blink rate during verbal recall in 21 medication-free children with ADHD and an increased blink rate during listening in 8 children with ADHD treated with stimulant medication compared to typically developing children. Interestingly, in comparison to control children, children with ADHD did not modulate blink rate across different cognitive tasks (listening, conversation, verbal recall), i.e., they showed smaller differences in blink rate between tasks. Because no generally reduced blink rates were observed, the authors conclude that other neurotransmitter systems like the noradrenergic system must be involved in the pathology of ADHD that cause the absence of blink rate modulation across tasks. Indeed, numerous pharmacological and biochemical studies suggest that both catecholamine systems [DA and noradrenaline (NE)] work less efficiently in ADHD, affecting a wide variety of higher control functions (top-down regulation of cognition, behaviour as well as emotion) (Arnsten and Pliszka 2011). The prefrontal networks involved in these control functions and the connectivity of these networks with other brain areas in particular are extremely sensitive to the neurochemical environment, with only small changes in DA and NE levels altering these functions significantly (Arnsten et al. 2010). Intact blink rates but decreased modulation of blink rates to task demands might therefore be suggestive of subtle suboptimal levels of DA and NE in the prefrontal cortex. This nicely fits the cognitive energetic model (CEM) of ADHD (Sergeant et al. 1999; Sergeant 2005), which makes a distinction between basic structural cognitive processes and energetic state processes (arousal, activation and effort) that modulate the structural processes. The CEM assumes that patients with ADHD have a deficient self-regulation of their energetic state, especially if a task is boring.

With these findings in mind, we hypothesized that patients with ADHD do not specifically suffer from reduced blink rates but rather show deficient modulation of blink rate to changing task demands, as a result of deficient self-regulation of their energetic state. To test this hypothesis, we extracted blink rate from an electrooculogram (EOG) that was measured in children with ADHD and healthy controls during a visual selective attention task and quiet rest. We expected no differences in basic blink rates, but reduced blink rate modulation from rest to task. In addition, we measured longer task duration than the previous studies (1 h instead of several minutes) to gain insight into time-on task effects for blink rate in ADHD. In line with recent findings in adults (Fried et al. 2014), we expected that children with ADHD have difficulties to maintain optimal levels of activation/arousal during task performance, resulting in a faster increment of increased blink rate with time-on task compared to controls. This tonic measure allows us to investigate the ability of these children to inhibit eye blinking for longer periods of task performance, i.e., to modulate blink rate. To gain insight into the effect of stimulant medication, which optimizes catecholamine levels in the prefrontal cortex (Arnsten and Pliszka 2011), we included a second ADHD group that was treated with individually tailored and clinically appropriate doses of methylphenidate (Mph) during the experiment. By the action of Mph on prefrontal control functions, we expect improved modulation of blink rate from rest to task and with time-on task.

Besides the conventional tonic measure of blink rate, we also explored a phasic measure of eye blink control in these clinical groups: the strategic timing of blinks relative to incoming visual information. The stimulus duration in the used paradigm was very short (lasting 100 ms), and therefore badly timed eye blinks (lasting 200–300 ms) during stimulus presentation could hamper information processing and accurate performance by temporarily blocking the visual system. We extracted the eye blinks from the EOG around the imperative stimuli with millisecond precision. A recent review on timing deficits in ADHD demonstrated that consistent impairments are found in motor timing, perceptual timing and temporal foresight comprising several timeframes spanning milliseconds, seconds, minutes as well as longer intervals up to years (Falter et al. 2013). We therefore expect that children with ADHD show reduced strategic timing of eye blinks around the imperative stimulus, hampering them from efficiently processing the incoming visual information. Mph might also have an improving effect on this phasic eye blink timing, because it has also been demonstrated to improve timing abilities in ADHD (Falter et al. 2013). Recent evidence from adults with ADHD indeed points to elevated blink rates during stimulus presentation, which is reduced, though not normalized, when these adults take stimulant medication (Fried et al. 2014).

## Methods

### Subjects

In this study we made use of the data collected during a previous electrophysiological study on the processing of reward and punishment in ADHD and the modulating effects of stimulant medication (Groen et al. 2009, 2013). We re-analysed the data and focussed on eye blink rate and timing. The study was approved by the Medical Ethical Committee of the University Medical Center Groningen, and written informed consent was obtained from all parents and all 12-year-old children. All procedures performed in studies involving human participants were in accordance with the ethical standards of the institutional and/or national research committee and with the 1964 Helsinki declaration and its later amendments or comparable ethical standards. Informed consent was obtained from all individual participants included in the study.

The study included fifty 10- to-12-year-old children belonging to three groups: a typically developing (TD) group (*n* = 18), a medication-free ADHD group (*n* = 16), and a Mph-treated ADHD group (*n* = 16). The TD children were recruited from primary schools in the city of Groningen and by advertisement in the newsletter of the University Medical Centre in Groningen (UMCG). The inclusion criteria for all children were: (1) 10–12 years of age, (2) a full-scale Intelligence Quotient (IQ) over 80 as measured by the Wechsler Intelligence Scale for Children-III (WISC-III), (3) right handed (or a tendency to right handedness). Handedness was measured by a self-report list (Van Strien 2003). None of the TD children had a formal or suspected psychiatric diagnosis. Additionally, the Child Behavioural Checklist which was filled out by the parents of all children (CBCL: Achenbach and Rescorla 2001), and none of the TD children scored within the clinical range of the total problem scale of the CBCL. See Table 1 for a summary of all group characteristics.

**Table 1:** Group characteristics

	TD (*n* = 18)	Mph-treated ADHD (*n* = 16)	Mph-free ADHD (*n* = 16)	*p* (*χ* ^2^)
	Ratio	Ratio	Ratio	
Handedness (ratio: left/ambidexter/right)	0/4/14	0/1/15	0/2/14	ns
Gender (ratio: male/female)	12/6	15/1	14/2	ns
Mph intake in past year (ratio: on/off)	0/18	15/1	12/4	<0.001, (TD < ADHD, ADHD Mph)

Measures	Mean (SD)	Mean (SD)	Mean (SD)	*p* (ANOVA)
Age (years)	11.4 (0.9)	11.4 (0.8)	11.7 (0.8)	ns
Total IQ	103 (9.5)	98 (11.3)	100 (13.4)	ns
Verbal IQ	107 (10.4)	100 (13.2)	102 (10.1)	ns
Performal IQ	97 (12.8)	96 (12.7)	98 (16.9)	ns
DISC attentional problems	–	12.6 (5.1)	12.9 (3.5)	ns
DISC hyperactive-impulsive behaviour	–	13.3 (3.3)	12.9 (5.2)	ns
CBCL total problems	14.8 (11.5)	47.8 (26.3)	59.8 (21.3)	<0.001 (TD < Mph-free, Mph-treated ADHD)
CBCL attentional problems	2.3 (2.1)	9.8 (3.5)	11.4 (1.7)	<0.001 (TD < Mph-free, Mph-treated ADHD)
CBCL internalizing problems	4.3 (4.4)	8.7 (8.0)	11.4 (8.5)	<0.05 (TD < Mph-free ADHD)
CBCL externalizing problems	3.5 (3.5)	13.3 (7.4)	17.6 (7.2)	<0.001 (TD < Mph-free, Mph-treated ADHD)
CTRS-R oppositional	–	59.3 (10.0)	58.9 (13.9)	ns
CTRS-R inattentive/cognitive problems	–	55.0 (8.1)	57.3 (13.6)	ns
CTRS-R Hyperactivity-Impulsivity	–	66.3 (9.4)	64.2 (14.4)	ns
CTRS-R anxious/shy	–	62.8 (13.5)	64.8 (11.4)	ns
CTRS-R perfectionism	–	56.1 (12.1)	53.3 (9.1)	ns
CTRS-R social problems	–	58.3 (9.0)	59.2 (15.4)	ns
CTRS-R ADHD index	–	63.8 (7.7)	63.7 (14.9)	ns

*TD* typically developing, *Mph* methylphenidate, *ADHD* attention deficit hyperactivity disorder, *DISC* Diagnostic Interview Schedule for Children, *CBCL* Child Behavioural Checklist, *CTRS-R* Conners’ Teacher Rating Scale-Revised

ADHD had been diagnosed by independent well-trained child psychiatrists of the Department of Child- and Adolescent Psychiatry, according to the diagnostic criteria of the DSM-IV-TR (American Psychiatric Association 2000). Only children with the combined type were included, which required pervasiveness (at home and at school) of both inattentive symptoms and hyperactive-impulsive symptoms observed during at least 6 months. Some of the symptoms caused impairment before age 7 years. The diagnosis was checked by administering the ADHD section of the Diagnostic Interview Schedule for Children-IV to the parents (DISC-IV: Shaffer et al. 2000; Dutch translation: Ferdinand and Van der Ende 1998) and the Conners’ Teacher Rating Scale-Revised (CTRS-R) to the teachers of the clinical children (Conners 1990, 1999). All children with ADHD scored in the clinical range of the DISC-IV ADHD section or in the borderline range of the CTRS-R. As 28 of the 30 children with ADHD were well-responding to Mph (2 children with ADHD were not taking medication at all), medication-intake in the period that was questioned by the interview likely caused underreport of ADHD symptoms. However, the Mph-treated and medication-free ADHD group did not differ in the number of symptoms as measured by the DISC-IV (see Table 1).

Of the 32 children with ADHD, 28 children were Mph responders, who all had taken this drug during the main part of the year preceding the experiment (except for one boy who had started the treatment 2 months before). The four children with ADHD that did not yet use medication for their ADHD symptoms were directly assigned to the medication-free condition. The 28 Mph responders were randomly assigned to the Mph-treated or medication-free condition. Those assigned to the medication-free condition were asked to delay their daily intake of medication until after the experiment. This resulted in a washout period of at least 17 h before the experiment, which is from a pharmacokinetic perspective sufficient for Mph to be completely metabolized into an inactive metabolite (5 times the half life time of extended release Mph and 8 times the half life time of immediate release Mph). Of the 32 children with ADHD, 13 children scored within the borderline or clinical range of the externalizing scale of the CBCL. This indicates that part of the children with ADHD may have had some symptoms of oppositional defiant disorder or conduct disorder, even though no formal comorbid psychiatric disorder was present. The medication-free and Mph-treated ADHD groups did not differ significantly in the severity of these externalizing problems (see Table 1).

### Task

#### Selective attention task

**Fig. 1:**
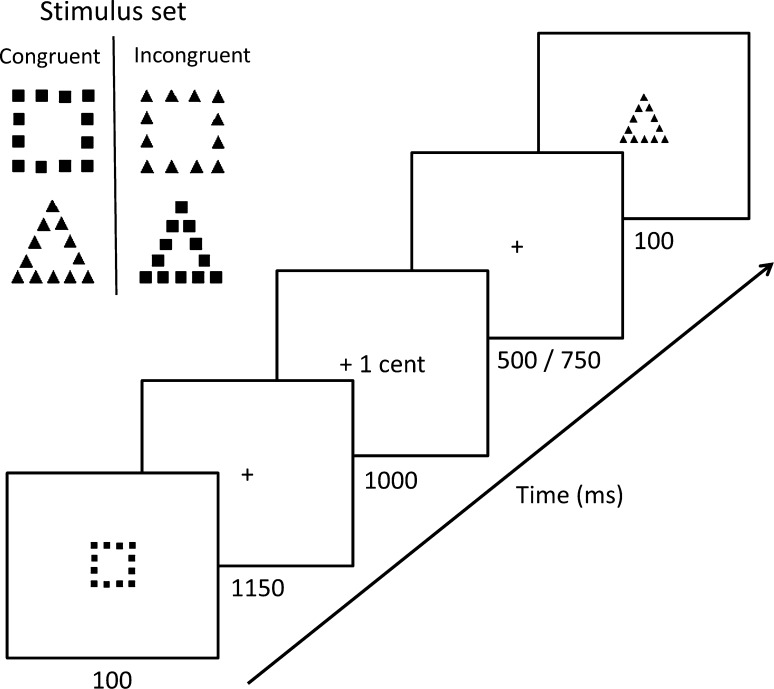
Example of a stimulus set and the sequence of events within a trial

Children performed a selective attention task with hierarchical stimuli, which was derived from the original Navon (1977) task and adapted for use in children. The task was built and presented with E-Prime (version 1.1; Psychological Software Tools). Three stimulus sets (see Fig. 1 for an example) were used that were alternated between feedback conditions (see below). In global blocks, the child had to press ‘left’ for the bigger ‘global’ triangles and ‘right’ for the bigger ‘global’ squares. In the local blocks, using the same stimulus set, the child had to press ‘left’ for the smaller ‘local’ triangles and ‘right’ for the smaller ‘local’ squares. Stimulus presentation was paced by the computer, but to take individual differences in response speed into account, an individual deadline time was imposed for every participant. The children performed six global and six local blocks, each consisting of 80 trials and lasting ~5 min, resulting in a total of 960 trials, with a total duration of ~60 min (see for more details Groen et al. 2009).

In the context of an individual response deadline, they were additionally instructed to respond accurately by encouraging them to earn as much money as possible in three feedback conditions: no feedback, win and loss. In the no feedback condition, the children received no information about the correctness of their response; each response was followed by a question mark. After finishing a no feedback block the children received 0.70 € independent of their performance. In the win condition the children started with 0.00 € and only correct responses resulted in a win of 0.01 €. Win and no win were indicated by ‘+1 c’ (in green) and ‘+0 c’ (in red) respectively. In the loss condition the children started with 0.80 € and only incorrect responses resulted in a loss of 0.01 €. Loss and no loss were indicated by ‘−1 c’ (in red) and ‘−0 c’ (in green) respectively. After every block the children received the money from the experimenter. Late reactions resulted in a penalty of 0.02 € in all feedback conditions.

### Procedure

The children were seated on a comfortable chair in front of a computer screen in a room that was separated from a control room by a one-way screen. After application of electrodes, the children started with a quiet rest block enduring 5 min. A second quiet rest block of 5 min was administered after completion of all experimental blocks. Between each block, a break of a few minutes was taken in which the child received payment. After six experimental blocks, there was a break of ~20 min. The instruction during quiet rest was to keep the eyes open during the rest blocks and fixate gaze at a drawing of a sleeping dragon. Unfortunately, roughly 1/3rd of the children were not able to fixate their gaze in a steady way during one or both of the rest blocks and those were instructed to close their eyes during the measurement. Rest measures for blink rate were therefore missing for these children (which were *n* = 6 for the TD group, *n* = 5 for the Mph-treated ADHD group and *n* = 3 for the Mph-free ADHD group).

#### EOG recordings and blink extraction procedure

EOG was recorded using Ag–AgCl electrodes, respectively, above and next to the left eye. Impedances were kept below 10 kΩ. Using the REFA-40 system (TMS International B.V.), the channels were amplified with filters set at a time constant of 1 s and a cut-off frequency of 130 Hz (low pass). The data from all channels were recorded with a sampling rate of 500 Hz using Portilab (version 1.10, TMS International B.V.).

**Fig. 2:**
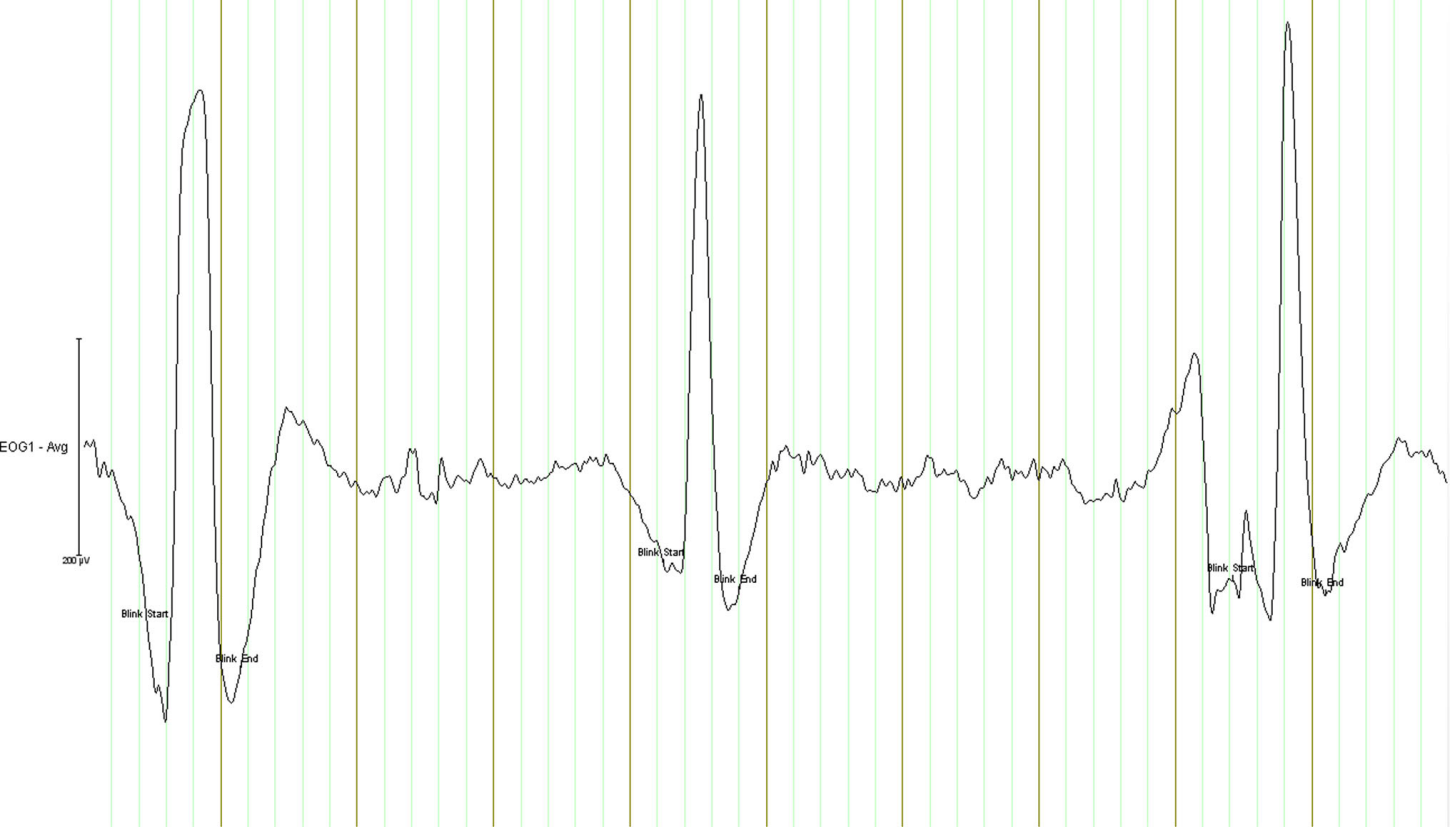
Example of several detected blinks in the electrooculogram of one child (*fat vertical lines* represent time periods of 1 s)

Using BrainVision 2 (Brain Products), the EOG signals were off-line filtered with a 1 Hz high pass and 20 Hz low pass filter, and referenced to the left ear electrode. The onset and offset of blinks were semi-automatically marked in the EOG by using the Independent Component Analysis blink detection algorithm. See Fig. 2 for an example of several detected blinks in one child. The blink rate for the rest and task blocks was established by a programme called ‘Blinkcounter’ which was developed by the Technical service of the Department of Psychology at the University of Groningen, The Netherlands. This programme counts the number of blinks in each task block and dividing this number by the duration of that block (in minutes). This resulted in a blink rate measure in blinks per minute (bpm) for each condition.

The timing of blinks around the imperative stimuli was investigated by examining the blink incidence before and after each stimulus presentation in an event-related fashion. The programme ‘Eyewink’ (which was again developed by the Technical service of the Department of Psychology) was used to count the number of blink onsets that occurred in six 100 ms-pre stimulus intervals (covering the ITI lasting 500 or 750 ms) and thirteen 100 ms-post stimulus intervals covering the presentation of both the stimulus enduring in the interval of 0–100 ms and the fixation cross lasting 1150 ms thereafter (which lasted until feedback onset) for each stimulus presentation in each condition. For each interval of 100 ms, the percentage of trials containing a blink was calculated with the following formula: (total number of blinks in a given interval/total number of trials) × 100 %.

#### Data analysis

The task performance of groups has been described previously in detail (Groen et al. 2009) and is shortly summarized in the present results section. The percentage of correct responses and correct RTs were analysed by means of a 3 × 2 × 3 mixed ANOVA design (SPSS version 16.0) with the within-subject variables ‘feedback’ (no feedback, gain and loss) and ‘level’ (global, local) and the between subjects factor ‘group’ (with the levels TD, ADHD Mph-free, and ADHD Mph-treated). In addition to Groen et al. (2009), time-on task effects on performance were investigated by computing the mean RT and percentage accurate for four quartiles of the total 1 h duration task. All quartiles consist of 3 successive task blocks (e.g., quartile 1 consists of the first 3 task blocks, which is ~15 min, quartile 2 consists of the second 3 task blocks, and so forth for quartiles 3 and 4). Time-on task effects were analysed with a repeated measures ANOVA with the within subjects factor ‘time-on-task’ (with the levels quartile 1, 2, 3, and 4) and the between subjects factor ‘group’ (with the levels TD, ADHD Mph-free, and ADHD Mph-treated).

Blink rate and blink rate modulation from rest to task was investigated with a 3 × 3 mixed ANOVA design with the within subjects factor ‘rest-task’ (with the levels rest and task) and the between subjects factor ‘group’ (with the levels TD, ADHD Mph-free, and ADHD Mph-treated). Secondly, blink rate modulation with time-on-task was investigated by computing the blink rate for four quartiles of the total task, which were analysed with a repeated measures ANOVA with the within subjects factor ‘time-on-task’ (with the levels quartile 1, 2, 3, and 4) and the between subjects factor ‘group’ (with the levels TD, ADHD Mph-free, and ADHD Mph-treated).

Blink timing around the stimulus was investigated by performing a 19 × 3 mixed ANOVA design, with the within subjects factor ‘interval’ (with the 19 intervals around the stimulus as levels) and the between subjects factor ‘group’ (with the levels TD, ADHD Mph-free, and ADHD Mph-treated). We adopted a hierarchical strategy of analysis, and performed separate ANOVAs for the 19 intervals to specify overall (interaction) effects with the factor ‘interval’. When testing effects in these 19 intervals, two consecutive intervals had to reach the α-level to be considered as meaningful, in order to correct for multiple testing. The chance of finding two consecutive effects with each showing a significance level of at least *p* = 0.05 in a series of 19 intervals is reduced to *p* = 18 × 0.05 × 0.05 = 0.045, which is below the significance criterion of *p* = 0.05. For inspecting the effects of time-on-task and feedback on blink timing, the analyses were repeated with adding the factors ‘quartile’ (with the levels quartile 1, 2, 3, and 4) and ‘feedback (with the levels no feedback, and feedback), respectively.

For all analyses, an α-level of <0.05 was adopted as the criterion of statistical significance. Greenhouse–Geisser adjusted *p* values are reported (and for valence effects the *ε*-correction factor), with the unadjusted degrees of freedom and *F* values. Partial eta squared effect sizes (*η* ^2^) were reported for the repeated measures analyses (small effects: *η* ^2^ < 0.06, medium effects: *η* ^2^ ≥ 0.06, and large effects *η* ^2^ ≥ 0.14). For between group comparisons, Cohen’s *d* effect sizes were calculated (negligible effects: *d* < 0.2, small effects: 0.2 < *d* < 0.5, medium effects: 0.5 < *d* < 0.8 and large effects: *d* > 0.8) (Cohen 1988).

## Results

### Task performance

A summary of the performance measures is presented. For the interested reader, more information is provided in Groen et al. (2009).

#### Accuracy and RT

The TD group performed significantly more accurately on the task than both the Mph-free and Mph-treated ADHD groups [main effect of group: *F*(2,47) = 3.4, *p* < 0.05, *η* ^2^ = 0.13]. All groups performed more accurately in the conditions with feedback than without feedback [main effect of feedback: *F*(2,94) = 14.4, *p* < 0.001, *η* ^2^ = 0.23], with the reward condition being slightly superior than the punishment condition (*p* < 0.01) in the TD and Mph-free ADHD group but not in the Mph-treated group. The groups did not differ in their mean RT (483 ms, SD 98 ms) (main effect of group: *p* > 0.05). The groups responded slower in the conditions with feedback than without feedback (main effect of feedback: *F*(2,94) = 6.3, *p* < 0.01, *η* ^2^ = 0.12, *ε* = 0.71), but no differences were found between groups (*p* > 0.05). All these effects did not differ between the global and local condition.

#### Time-on task effects on accuracy and performance

**Fig. 3:**
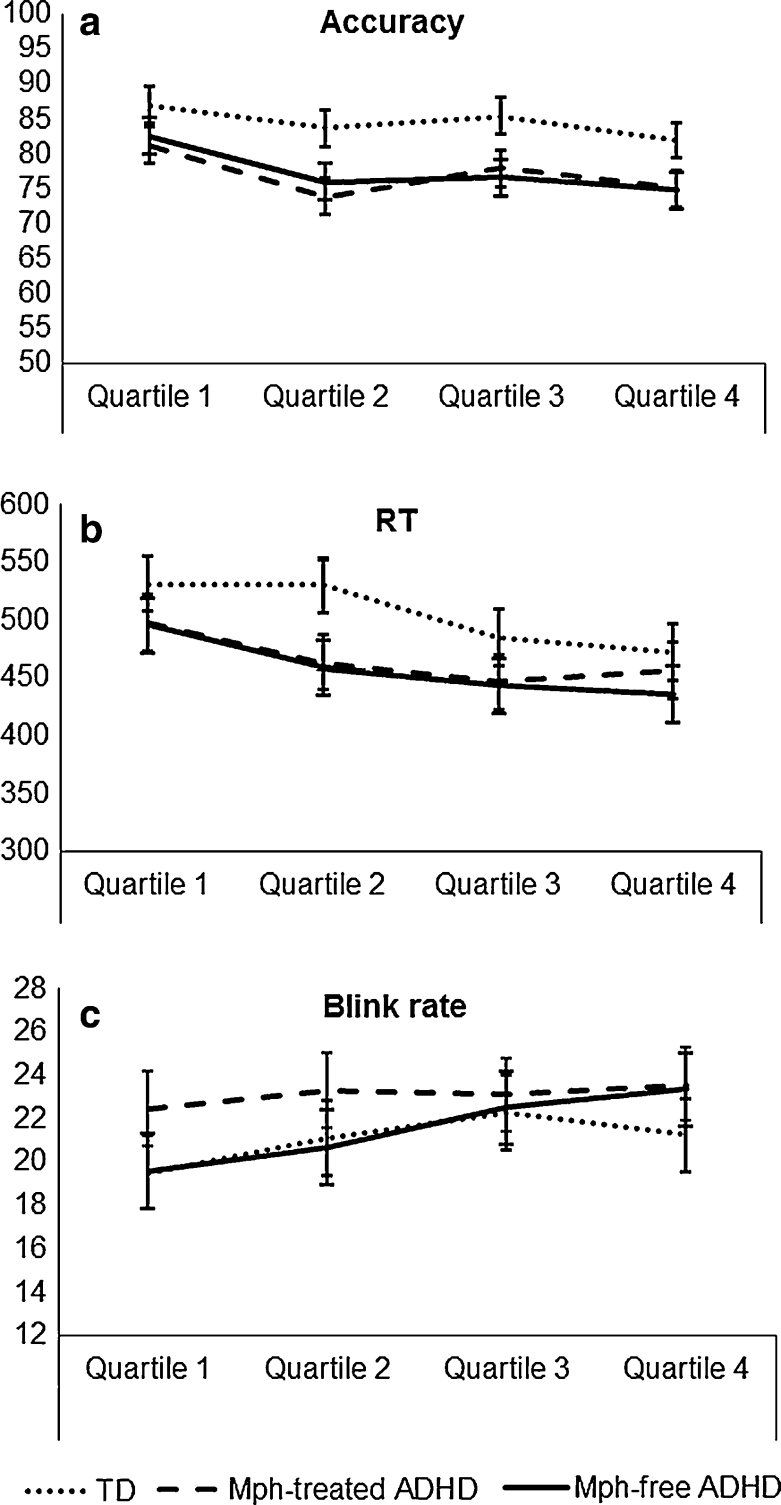
Time-on-task effects on **a** accuracy, **b** reaction time (RT), and **c** blink rate, separated by group (*error bars* reflect standard errors)

As can be seen in Fig. 3, accuracy decreased with time-on task [main effect of time-on task: *F*(3,141) = 11.0, *p* < 0.001, *η* ^2^ = 0.19] from quartile 1 to 2 (*p* < 0.001), and with a trend to significance from quartile 2 to 3 (*p* = 0.076) and quartile 3 to 4 (*p* = 0.056). The groups did not differ from each other in this time-on task effect [*F*(6,141) = 0.65, *p* > 0.05, *η* ^2^ = 0.03], and for none of the contrasts. RT also decreased with time-on task [main effect of time-on task: *F*(3,141) = 16.1, *p* < 0.001, *η* ^2^ = 0.26], from quartile 1 to 2 (*p* < 0.001) and quartile 2 to 3 (*p* < 0.01). Although the groups did not differ in the overall time-on task effect for RT [*F*(6,141) = 1.2, *p* > 0.05, *η* ^2^ = 0.05], contrasts between the quartiles revealed that the groups differed in the reduction of RT from quartile 1 to 2 [*F*(1,47) = 6.2, *p* < 0.01, *η* ^2^ = 0.21). Post hoc analyses between the groups indicated that both ADHD groups showed a reduction in RT for this contrast whereas the TD group did not.

### Blink rate during rest and task

**Fig. 4:**
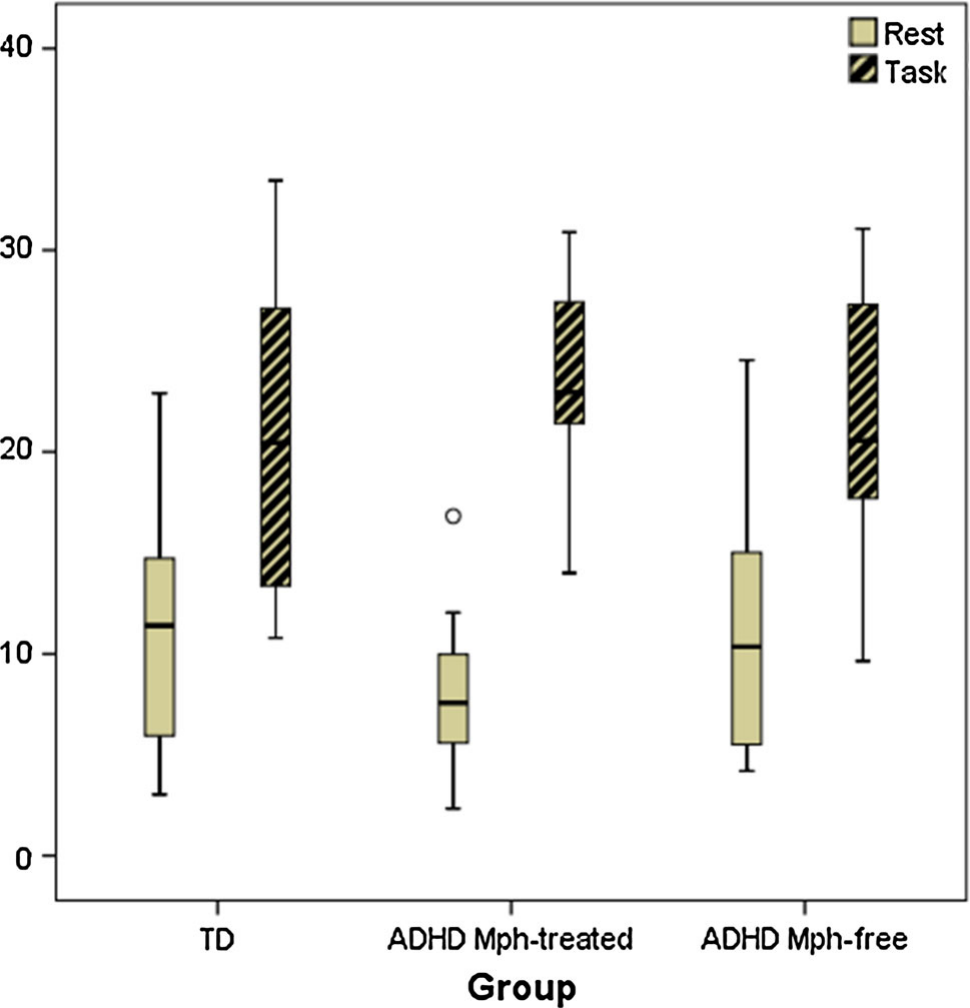
*Box plots* of blink rate (bpm) for quiet rest and task performance, separated by group

The blink rate increased from rest to task in all groups with large effect size, see Fig. 4 [main effect of ‘task-rest’: *F*(1,33) = 83.8, *p* < 0.001, *η* ^2^ = 0.72]. The groups did not differ significantly in this effect, but the difference between groups was of medium size [non-significant interaction effect of ‘task-rest’ × group: *F*(2,33) = 2.1, *p* = 0.14, *η* ^2^ = 0.11]. Across rest and task conditions, there was no group difference in overall blink rate [non-significant main effect of ‘group’: *F*(2,33) = 0.10, *p* = 0.91, *η* ^2^ = 0.01]. Separate analyses for rest and task did also not reveal group differences in blink rate [non-significant main effect of ‘group’ for rest: *F*(2,35) = 1.3, *p* = 0.30, *d* = 0.68 and for task: *F*(2,47) = 0.4, *p* = 0.64, *d* = 0.54].

### Blink rate during time-on-task

The blink rate increased with time-on-task for all groups with large effect size, see Fig. 3c [main effect of ‘time-on-task’: *F*(3,141) = 9.2, *p* < 0.001, *η* ^2^ = 0.16]. The blink rate increased during the first 45 min of the task and became steady during the last 15 min. This was demonstrated by significant repeated contrasts for quartile 1–2 [*F*(1,47) = 10.3, *p* < 0.01, *η* ^2^ = 0.18], quartile 2–3 [*F*(1,47) = 5.9, *p* < 0.05, *η* ^2^ = 0.11], but not from quartile 3–4 [*F*(1,47) = 0.07, *p* < 0.80, *η* ^2^ = 0.01]. The groups did not differ significantly in the time-on-task effect [non-significant interaction effect of ‘time-on-task’ × group: *F*(6,141) = 1.8, *p* = 0.13, *η* ^2^ = 0.07].

As the groups performed consistently less accurate and faster in the task conditions without feedback as compared to the feedback conditions, further analysis was performed in order to examine whether blink rate differed between these conditions and whether this influenced time-on task effects. Feedback condition (with and without feedback) had no effect on blink rate [*F*(1,47) = 0.04, *p* = 0.84, *η* ^2^ = 0.00], but interacted with ‘group’ [*F*(2,47) = 3.6, *p* < 0.05, *η* ^2^ = 0.13]. Analyses by group revealed that only the Mph-treated ADHD group showed an increased blink rate in the condition with feedback compared to no feedback [Mph-treated ADHD group: *F*(1,15) = 4.7, *p* < 0.05, *η* ^2^ = 0.24; other groups *p* >0.05). Post hoc group comparisons demonstrated that this difference found in the Mph-treated ADHD group differed significantly from the TD group [*F*(1,32) = 7.3, *p* < 0.05, *η* ^2^ = 0.19], and showed a trend towards a difference in the Mph-free ADHD group [*F*(1,30) = 3.1, *p* = 0.09, *η* ^2^ = 0.10].

The interaction of feedback condition with time-on-task effects was investigated with two task sections (section 1 = quartile 1 + quartile 2, and section 2 = quartile 3 + quartile 4), because computation of the quartiles separated for each feedback condition resulted in many missing values resulting from the random presentation order of the feedback conditions (e.g., in some participants the ‘no feedback’ condition was not present in quartile 1). No significant influence of feedback condition on time-on-task (sections 1, 2) effect was found [*F*(1,47) = 3.6, *p* = 0.06, *η* ^2^ = 0.07], and neither did group interact with this effect [F(2,47) = 0.1, *p* = 0.91, *η* ^2^ = 0.00]. Inspection of data indicated that the trend to significance of the ‘time-on-task’ × ‘feedback condition’ interaction pointed in all groups to a slightly increased time-on-task effect in the conditions without feedback compared to the conditions with feedback.

### Blink timing around the imperative stimulus

**Fig. 5:**
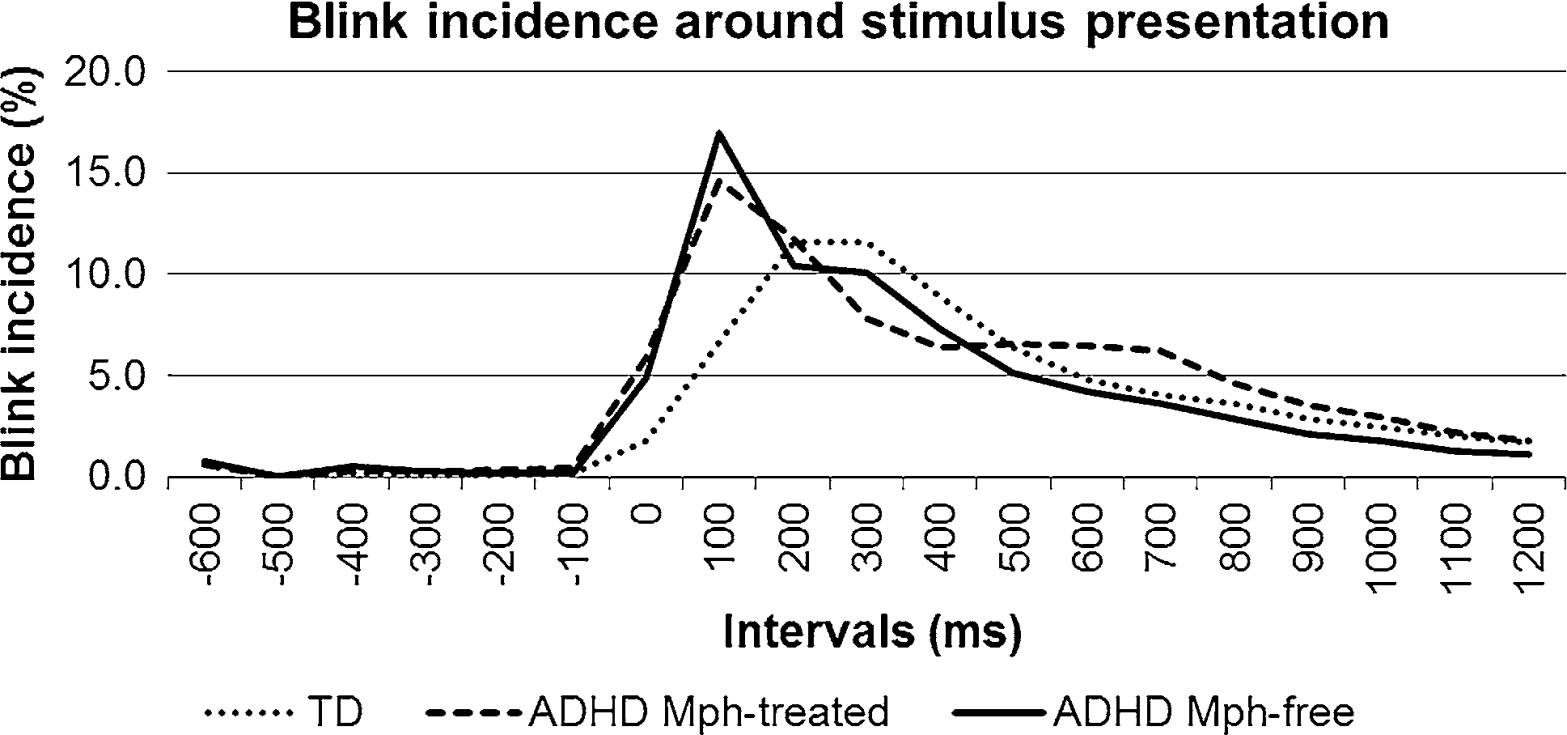
Blink incidence (in percentage of the trials containing a blink) around the imperative stimulus, separated by group

The incidence of blinks (i.e., start of blink activity) was near zero before stimulus onset, increased gradually during stimulus presentation to 3–5 %, peaked to 11–17 % in the two intervals after stimulus offset, and decreased gradually towards the feedback onset (see Fig. 5). This was reflected by a significant effect of interval which was of large effect size [*F*(18,846) = 20.2, *p* < 0.001, *η* ^2^ = 0.30, *ε*_GG_ = 0.15]. In the main analysis, this pattern did not differ between groups as indicated by absence of an ‘interval’ × ‘group’ interaction [*F*(18,836) = 1.3, *p* = 0.37, *η* ^2^ = 0.04, *ε*_GG_ = 0.15]. Polynomial contrasts, however, pointed to significance of the linear contrast [*F*(2,47) = 3.3, *p* < 0.05, *η* ^2^ = 0.12], but quadratic and cubic contrasts were non-significant. Separate ANOVAs were calculated for each interval in order to explore the significant contrast and revealed that only two successive intervals reached the set α-level criterion for group differences, i.e., the intervals −500 and −400 [−500: *F*(2,49) = 6.3, *p* < 0.01; −400: *F*(2,49) = 4.6, *p* < 0.05]. In these intervals, the Mph-free ADHD group showed roughly twice as much blinks than the TD group [−500: *F*(1,32) = 7.2, *p* < 0.05; −400: *F*(1,32) = 8.3, *p* < 0.01], but blink incidence levels still did not exceed 1 % of the trials. Surprisingly, the group differences in the intervals 0 and 100 as suggested in Fig. 5 were not significant [0: *F*(2,49) = 0.7, *p* = 0.51, *d* = 0.56; 100: *F*(2,49) = 1.5, *p* = 0.23, *d* = 0.56].

In line with the above results, a direct comparison of the prestimulus blink latencies (in the −600 to 0 ms interval) and the poststimulus blink latencies (in the 0–1250 ms interval) revealed no group differences [prestimulus: *F*(2,49) = 0.3, *p* = 0.75, *d* = 0.60; poststimulus: *F*(2,49) = 0.5, *p* = 0.63, *d* = 0.60].

Adding the factor ‘feedback condition’ (no feedback, feedback) to the interval analysis revealed that ‘feedback condition’ influenced the blink incidence pattern around the stimulus significantly [interaction ‘feedback condition’ × ‘interval’: *F*(18,846) = 5.2, *p* < 0.001, *η* ^2^ = 0.10, *ε*_GG_ = 0.31]. Analyses per interval indicated that the incidence of eye blinks in the no feedback condition was higher than in the conditions with feedback in the intervals 100–300 ms [100: *F*(1,47) = 4.6, *p* < 0.05, *η* ^2^ = 0.09; 200: *F*(1,47) = 14.0, *p* < 0.001, *η* ^2^ = 0.23; 300: *F*(1,47) = 6.0, *p* < 0.05, *η* ^2^ = 0.11], and from 1000 to 1200 ms [1000: *F*(1,47) = 9.1, *p* < 0.01, *η* ^2^ = 0.16; 1100: *F*(1,47) = 15.2, *p* < 0.001, *η* ^2^ = 0.25; 1200: *F*(1,47) = 6.8, *p* < 0.05, *η* ^2^ = 0.13]. No significant three-way interaction of ‘interval’ × ‘feedback condition’ × ‘group’ was present [*F*(36,846) = 1.4, *p* = 0.18, *η* ^2^ = 0.06, *ε*_GG_ = 0.31].

Adding the factor ‘time-on task’ (quartile 1–4) to the interval analysis revealed no significant influence of time-on task on the blink incidence pattern around the stimulus [interaction ‘time-on task’ × ‘interval’: *F*(54,2538) = 1.6, *p* = 0.13, *η* ^2^ = 0.003, *ε*_GG_ = 0.12]. Repeated contrasts of the quartiles indicated that there was only for the comparison of quartile 1–2 a quadratic difference between the feedback and no feedback condition [(*F*(1,47) = 4.3, *p* < 0.05, *η* ^2^ = 0.10], which is indicative of a reduced blink incidence around the stimulus in quartile 1. This interaction did not differ between groups [interaction ‘time-on task’ × ‘interval’ × ‘group’: *F*(108,2538) = 1.7, *p* = 0.07, *η* ^2^ = 0.07, *ε*_GG_ = 0.12] and no significant contrasts were found for the ‘time-on task’ × ‘interval’ × ‘group’ interaction.

## Discussion

This study investigated the spontaneous eye blink rate and strategic timing of eye blinks around the imperative stimuli during a visual selective attention task in children with ADHD who were on or off stimulant medication. In contrast to our hypotheses, the results indicate that there are neither differences in spontaneous blink rate during rest and task, nor in the modulation of blink rate to the task demands between TD children and children with ADHD that are off medication. Both Mph-free children with ADHD and TD children increased their blink rate from rest to task, showed no blink rate modulation to the presence of performance feedback during the task, and showed an increased blink rate with time-on-task paralleling their performance decrement. This performance decrement, i.e., a reduced accuracy and increased speed with time-on-task, did also not differ between the groups. Mph treatment also did not have the expected overall effect of increasing the blink rate during rest and task, and neither to increase modulation of blink rate from rest to task. However, different from the TD children and Mph-free children with ADHD it appeared that the Mph-treated ADHD group showed an increased blink rate in conditions with performance feedback, compared to conditions without feedback (large effect size). Furthermore, explorative analyses of blink timing around the imperative stimuli in the selective attention task revealed that in all three groups eye blinks are inhibited before stimulus onset, when the stimulus is expected to occur, and released after stimulus onset. The Mph-free ADHD group showed reduced eye blink inhibition between 300 and 500 ms before stimulus onset. However, this reduced blink inhibition in children with ADHD off medication is not likely to be detrimental for stimulus intake, as will be discussed below.

The finding of normal blink rates in children with ADHD accumulates to a series of studies reporting no reduced blink rates in children with ADHD (Caplan et al. 1996; Daugherty et al. 1993; Jacobsen et al. 1996; Tantillo et al. 2002). To date, only one study reported a significantly reduced blink rate in children with ADHD (Konrad et al. 2003), whereas the only study in adults with ADHD reported a significantly enhanced blink rate (Fried et al. 2014). Assuming that blink rate is a measure for central DA- function, blink rate studies so far provided only limited evidence for theories suggesting a hypodopaminergic state in ADHD (Levy and Swanson 2001; Oades et al. 2005). Numerous neuroimaging, pharmacological and animal studies have, however, found support for a ‘relative’ hypodopaminergic state in anterior frontostriatal systems (Levy and Swanson 2001), but it should be stressed that this hypodopaminergic reflects a balance between subcortical and cortical areas and a balance with other neurotransmitters such as NE (Arnsten and Pliszka 2011; Castellanos 1997). Even a delicate imbalance in these neurotransmitters can cause altered prefrontal functions, resulting in impaired executive functions, including working memory and inhibition problems that are often observed in ADHD. Although blink rate may be sensitive to more fundamental disturbances in the nigrostriatal system, such as in Parkinson’s disease and schizophrenia (Deuschl and Goddemeier 1998; Karson et al. 1981; Karson 1983; Kleinman et al. 1984), we suggest that it may not be a stable and sensitive measure for more delicate imbalances in the DA frontostriatal system as are observed in ADHD.

The finding of normal blink modulation to task demands (from rest to task) and to internal state (during performance decrement with time-on task) in children with ADHD is not in line with the results of Caplan et al. (1996) who demonstrated a reduced eye blink modulation across different tasks in children with ADHD. A possible explanation for the divergent findings is the application of different types of tasks between the studies; we performed a resting condition and a visual selective attention task, whereas Caplan and colleagues used listening, conversation and verbal recall tasks. In the light of the CEM, it could well be the case that the modulation of eye blink rates in ADHD is dependent on the event rates used in the task at hand. Previous studies (Metin et al. 2012; van der Meere et al. 2009) suggest that children with ADHD perform optimally in tasks with intermediate event rates (inter-stimulus intervals of 3–5 s), and perform more poorly on long, boring tasks with slow event rates (inter-stimulus intervals ≥6 s) or over stimulating tasks with fast event rates (inter-stimulus intervals ≤2 s). A neuroimaging study indeed found pronounced abnormal frontostriatal brain activation in adults with ADHD while they performed a go-nogo task with slow event rate and relatively normalized activation with a fast event rate (Kooistra et al. 2010). Therefore, differences in blink rate between children with and without ADHD might particularly be found on tasks with either very slow or very fast event rates. The event rate of the stimuli in the visual selective attention task used in the present study was ~3 s which can be regarded as a rather fast event rate. Interestingly, Fried et al. (2014) found enhanced blink rates using an attention task with a fast event rate (~2 s). Given these inconsistent findings, future studies on blink rate and blink rate modulation in individuals with ADHD should systematically vary event rates in their tasks. Furthermore, also the use of fixed versus variable inter-stimulus intervals was mentioned to be of importance, as only regular intervals appeared to induce group differences (Fried et al. 2014).

Another relevant aspect when studying blink rates in ADHD is sleepiness, especially because sleep problems are common in patients with ADHD (Cortese et al. 2009). Previous research found that sleep deprivation induces increased blink rates in healthy adults (Barbato et al. 2007). This effect was interpreted as an increase of dopamine activation following sleep deprivation, which allows the participant to fight the sleep. Since the present study found no elevated blink rates in children with ADHD, we have no reason to suspect that increased sleepiness in the children with ADHD influenced the results. The increase of blink rates with increased time-on task does likely reflect an increased level of fatigue (Stern et al. 1994); however, these effects were also similar between groups.

In contrast to our expectations, the blink rates were generally not different for the children with ADHD that were on stimulant medication during the experiment compared to those that were off medication. Interestingly, however, the children with ADHD on Mph increased their blink rates during the conditions with performance feedback compared to those without feedback, whereas this was not the case for the TD and Mph-free ADHD group. The combination of Mph and performance feedback may therefore increase blink rates and therefore increase midbrain DA-activity. We speculate that this effect is caused by a specific enhancing effect of Mph of punishment sensitivity which was also observed in both the accuracy of performance and the evoked heart rate activity to negative feedback that were specifically enhanced in the feedback condition with punishing feedback (Groen et al. 2009). Stimulant medication in ADHD may increase motivation to perform well under threat of punishment which is reflected by enhanced midbrain DA-activity and blink rates.

In this study, an explorative analysis was performed on the strategic timing of eye blinks around the imperative stimuli during the visual attention task. As the stimulus duration was only 100 ms, eye blinks positioned during stimulus presentation would hamper information processing and accurate performance. In line with previous studies (Pivik and Dykman 2004; Sirevaag et al. 1999), children inhibited their eye blinks before and during the stimulus and released eye blinks after the stimulus. In the period of 600 ms before the stimulus, in which children were awaiting the stimulus presentation, the blink incidence was near zero in all groups. Only in the Mph-free children with ADHD the blink incidence was significantly enhanced to ~1 % in the period of 300–500 ms before the stimulus onset. Although this is an indication that Mph-free children with ADHD show reduced blink inhibition when awaiting a salient stimulus, this is not likely to impair their visual information processing and performance accuracy because these blinks were extremely infrequent (<1 % of the trials) and were timed well before stimulus onset so that no important information would be missed. In all groups, the majority of blinks were timed after the stimulus presentation and no differences were found between the TD group, the Mph-free ADHD group and the Mph-treated ADHD group. This demonstrates that on the millisecond level, there are no timing deficits in the positioning of eye blinks around the stimulus. This finding is not in line with a recent review demonstrating consistent impairments in motor timing, perceptual timing and temporal foresight comprising several timeframes, and that Mph may improve these abilities (Falter et al. 2013). However, one critical factor influencing these results could be the use of a variable inter-stimulus interval in the present study. In the study by Fried et al. (2014), adults with ADHD did position their eye blinks more often during the stimulus compared to controls in a task using a fixed inter-stimulus interval. This result, however, was not obtained when using a variable inter-stimulus interval. The authors suggested that fixed timing therefore may have a critical influence on the positioning of eye blinks and differences between individuals with and without ADHD.

### Limitations

The results of this study have to be interpreted in the light of some study limitations. First of all, the sample size was rather small (*n* = 16 Mph-free ADHD, *n* = 16 Mph-treated ADHD, and *n* = 18 TD), which may have caused that small or medium effects were missed because of insufficient statistical power. Post hoc power analyses, however, indicated that the effect sizes for the blink rate differences between the Mph-free ADHD and TD group were near zero (0.00 < *d* < 0.12), indicating that no group differences can be expected with increasing sample sizes. Moreover, the increased blink incidence during the stimulus in the ADHD groups as suggested by Fig. 5, appeared to be of medium effect size. Post hoc power analyses showed that these effects would only reach significance if more than 674 children are included in each group (repeated measures analysis). An Mph effect on blink rate may, however, be demonstrated when increasing sample size. The effect sizes for blink rate differences between the Mph-treated and TD group appeared to be small for an increased blink rate during task (*d* = 0.41), medium for a decreased blink rate during rest (*d* = 0.58), and large for an enhanced blink rate increase from rest to task (*d* = 0.80), indicating that samples ranging from *n* = 48 to 95 subjects in each group may demonstrate significant Mph-effects on blink rate in children with ADHD. Thus, the null findings for the Mph-free and TD comparison are not likely to be the result of low statistical power, whereas increasing the sample sizes substantially could elicit Mph-effects on blink rates.

In this study, Mph-effects were investigated using a between subjects design. Future studies on Mph-effects on blink rate should make use of a double-blind placebo-controlled cross-over design for allowing more firm conclusions. Furthermore, a relatively short washout period was chosen in order to reduce the burden for patients. Although the used period of at least 17 h is from a pharmacokinetic perspective sufficient for complete washout, the compound may have exerted neurobiological effects and reduced potential group differences. However, using a similar setup, a recent study in adults did find within-subject group differences on and off medication for blink rates (Fried et al. 2014). We therefore do not believe that this is a major concern for the interpretation of the current findings.

## Conclusions

Using a visual selective attention task with a fast event rate (inter-stimulus interval ~3 s) and a rest condition, blink rate and blink timing was investigated in Mph-free and Mph-treated children with ADHD. No evidence was found for aberrant blink rate and blink modulation in children with ADHD off Mph (with near zero effect sizes). Stimulant medication appeared not to influence blink rate and blink modulation, except for an enhancement of blinks when performance feedback was provided. All groups strategically timed their blinks after stimulus presentation. Mph-free children with ADHD showed reduced blink inhibition before the stimulus; however, given the low incidence (<1 %) and long latency this is not likely to impair their visual intake. Future studies should replicate these results while systematically varying different event rates and fixed versus variable inter-stimulus intervals, as well as performing within subjects measurements of Mph-effects.
